# The minimally effective dose of sucrose for procedural pain relief in neonates: a randomized controlled trial

**DOI:** 10.1186/s12887-018-1026-x

**Published:** 2018-02-23

**Authors:** Bonnie Stevens, Janet Yamada, Marsha Campbell-Yeo, Sharyn Gibbins, Denise Harrison, Kimberley Dionne, Anna Taddio, Carol McNair, Andrew Willan, Marilyn Ballantyne, Kimberley Widger, Souraya Sidani, Carole Estabrooks, Anne Synnes, Janet Squires, Charles Victor, Shirine Riahi

**Affiliations:** 1 The Hospital for Sick Children, Lawrence S. Bloomberg Faculty of Nursing, University of Toronto, 686 Bay Street, Toronto, Ontario M5G 0A4 Canada; 2 Daphne Cockwell School of Nursing, Ryerson University, 350 Victoria Street, Toronto, Ontario M5B 2K3 Canada; 3 School of Nursing and Departments of Pediatrics, Psychology, and Neuroscience, Dalhousie University, IWK Health Centre, Forrest Building, P.O. Box 15000, Halifax, Nova Scotia B3H 4R2 Canada; 4 Trillium Health Partners, 100 Queensway West, Mississauga, Ontario L5B 1B8 Canada; 5 Faculty of Health Sciences, School of Nursing, University of Ottawa, Children’s Hospital of Eastern Ontario, Research Institute, 451 Smyth Road, Ottawa, Ontario K1H 8M5 Canada; 6 The Hospital for Sick Children, 686 Bay Street, Toronto, Ontario M5G 0A4 Canada; 7 The Hospital for Sick Children, Leslie Dan Faculty of Pharmacy, University of Toronto, 686 Bay Street, Toronto, Ontario M5G 0A4 Canada; 8 The Hospital for Sick Children, 686 Bay Street, Toronto, Ontario M5G 0A4 Canada; 9 The Hospital for Sick Children, Dalla Lana School of Public Health, University of Toronto, 686 Bay Street, Toronto, Ontario M5G 0A4 Canada; 10 Holland Bloorview Kids Rehabilitation Hospital, Lawrence S. Bloomberg Faculty of Nursing, University of Toronto, 150 Kilgour Road, Toronto, Ontario M4G 1R8 Canada; 11 The Hospital for Sick Children, Lawrence S. Bloomberg Faculty of Nursing, University of Toronto, 155 College Street, Suite 130, Toronto, Ontario M5T 1P8 Canada; 12 Daphne Cockwell School of Nursing, Ryerson University, 350 Victoria Street, Toronto, Ontario M5B 2K3 Canada; 13 Faculty of Nursing, University of Alberta, 3-141 Edmonton Clinic Health Academy, 11405 87 Avenue, Edmonton, Alberta T6G 1C9 Canada; 14 Division of Neonatology, Department of Pediatrics, University of British Columbia, 2D19-4480 Oak Street, Vancouver, British Columbia V6H 4V4 Canada; 15 Faculty of Health Sciences, School of Nursing, University of Ottawa, Ottawa Hospital Research Institute, 451 Smyth Road, Ottawa, Ontario K1H 8M5 Canada; 16 Institute for Clinical Evaluative Sciences (ICES), The Institute of Health Policy, Management and Evaluation, University of Toronto, Veterans Hill Trail, 2075 Bayview Avenue G1 06, Toronto, Ontario M4N 3M5 Canada; 17 The Hospital for Sick Children, 686 Bay Street, Toronto, Ontario M5G 0A4 Canada

**Keywords:** Adverse event, Analgesia, Heel lance, Neonates, NICU, Pain, PIPP-R, Preterm infants, Sucrose

## Abstract

**Background:**

Orally administered sucrose is effective and safe in reducing pain intensity during single, tissue-damaging procedures in neonates, and is commonly recommended in neonatal pain guidelines. However, there is wide variability in sucrose doses examined in research, and more than a 20-fold variation across neonatal care settings. The aim of this study was to determine the minimally effective dose of 24% sucrose for reducing pain in hospitalized neonates undergoing a single skin-breaking heel lance procedure.

**Methods:**

A total of 245 neonates from 4 Canadian tertiary neonatal intensive care units (NICUs), born between 24 and 42 weeks gestational age (GA), were prospectively randomized to receive one of three doses of 24% sucrose, plus non-nutritive sucking/pacifier, 2 min before a routine heel lance: 0.1 ml (Group 1; *n* = 81), 0.5 ml (Group 2; *n* = 81), or 1.0 ml (Group 3; *n* = 83). The primary outcome was pain intensity measured at 30 and 60 s following the heel lance, using the Premature Infant Pain Profile-Revised (PIPP-R). The secondary outcome was the incidence of adverse events. Analysis of covariance models, adjusting for GA and study site examined between group differences in pain intensity across intervention groups.

**Results:**

There was no difference in mean pain intensity PIPP-R scores between treatment groups at 30 s (*P* = .97) and 60 s (*P* = .93); however, pain was not fully eliminated during the heel lance procedure. There were 5 reported adverse events among 5/245 (2.0%) neonates, with no significant differences in the proportion of events by sucrose dose (*P* = .62). All events resolved spontaneously without medical intervention.

**Conclusions:**

The minimally effective dose of 24% sucrose required to treat pain associated with a single heel lance in neonates was 0.1 ml. Further evaluation regarding the sustained effectiveness of this dose in reducing pain intensity in neonates for repeated painful procedures is warranted.

**Trial registration:**

ClinicalTrials.gov: NCT02134873. Date: May 5, 2014 (retrospectively registered).

## Background

Multiple trials and recent systematic reviews with meta-analyses have shown that sweet solutions, including orally administered sucrose, are effective and safe in reducing pain intensity (using clinical observational or composite measures) during single, tissue-damaging procedures in neonates [1, 2]. These solutions are commonly recommended in neonatal pain guidelines [3]. However, there is wide variability in sucrose doses examined in research, and more than a 20-fold variation across neonatal care settings [4]. Despite the large number of randomized controlled trials in the 2016 Cochrane review [2], an optimal dose of sucrose could not be determined due to the wide range of volumes and concentrations (0.05 ml of 24% to 2.0 ml of 50% solution) studied, and due to variation in study methods (e.g., administration techniques, types of painful procedures, outcome measures, and co-interventions). There are no definitive conclusions about the minimally effective dose of sucrose associated with a clinically significant reduction in pain intensity scores in neonates.

To our knowledge, there have been no direct comparisons of different volumes of sucrose at the same concentration. In this study, we evaluated the three smallest doses of sucrose most commonly reported to be effective in previous research (i.e., 0.1 ml, 0.5 ml, and 1.0 ml of 24% sucrose) [2] to determine the minimally effective dose for neonates undergoing a skin-breaking heel lance procedure while in the neonatal intensive care unit (NICU). Doses smaller than 0.1 ml were not included in the study due to challenges posed by accurate measurement and delivery. All neonates received sucrose for procedural pain (i.e., there was no placebo or no-treatment group), which was consistent with neonatal pain guidelines and in keeping with the ethical conduct of clinical trials in newborns [5–7]. We hypothesized that (a) there was no difference in pain intensity between the sucrose doses, measured at 30 and 60 s following the heel lance using the Premature Infant Pain Profile-Revised (PIPP-R), and (b) adverse events would be minimal.

## Methods

A prospective multi-centered single-blind randomized controlled trial was conducted from July 2013–April 2015 at 4 Canadian tertiary NICUs following research ethics approval. The inclusion criteria were neonates 24 to 42 weeks gestational age (GA) at birth and less than 30 days of life/or less than 44 weeks GA at the time of the intervention, scheduled to receive a heel lance, and who had not received opioids within 24 h prior to the heel lance. The exclusion criteria were neonates with a contraindication for sucrose administration (e.g., were too ill or unstable as per neonatologist’s assessment, unable to swallow, pharmacologically muscle relaxed) and/or inability to assess behavioral responses to pain accurately (e.g., the neonate’s face was blocked with taping). We did not use the diagnosis of neurological impairment as an exclusion criterion because the timing of diagnosis and determining the severity of impairment can be very difficult in this population. However, inability to swallow had the effect of excluding neonates with severe neurologic impairment from hypoxic-ischemic encephalopathy. Observation of the procedure was timed to ensure that no additional sucrose doses were provided within the previous 4 h. All parents or legal guardians provided informed consent.

Randomization was performed using a web-based privacy protected randomization service [8]. Randomization was block stratified by GA at birth (< 29 weeks or 29–42 weeks) to enhance balanced intervention groups. A research nurse, aware of group allocation, drew up the assigned sucrose dose into an amber colored syringe. The dose was double-checked by a second nurse, not involved with the study, and documented on the medication administration record as per unit protocol. The research nurse followed a standard dose administration time to blind the bedside nurse performing the heel lance to the sucrose volume. The syringes used to administer sucrose were also shielded from view by the research nurse from the bedside nurse and video recording. No other study personnel had access to the treatment allocation.

The treatment intervention was videotaped and included 4 phases. (a) Baseline observation of the neonate for 2 min prior to the heel lance. (b) Administering the total volume of 24% sucrose [0.1 ml(Group 1), 0.5 ml (Group 2), or 1.0 ml (Group 3)] drop-by-drop via syringe over the anterior surface of the tongue, allowing for individual neonate swallowing rates over a period of 1–2 min (for the largest dose). A pacifier was offered to all neonates immediately following sucrose administration to facilitate non-nutritive sucking, which has been shown to enhance sucrose efficacy in a synergistic way [9]. (c) Conducting the heel lance procedure with an automated lancet approximately 2 min after the sucrose administration, to allow for peak effects [10]. (d) Observation of return-to-baseline pain indicator values over 30 s to several minutes. The bedside nurse conducted the heel lance according to the specific unit policy, while the research nurse experienced in NICU care ensured complete data collection.

We did not limit participating neonates from receiving other pain-relieving parent-initiated interventions (e.g., skin-to-skin/kangaroo care and breastfeeding) [11] as per unit protocols. These were documented by the research nurse, so any group differences could be controlled for in the analysis. Pharmacological interventions shown to be ineffective in reducing heel lance pain (e.g., acetaminophen) [12] were not administered.

### Outcome measures

The primary outcome was pain intensity measured with the PIPP-R [13, 14], which has demonstrated construct validity in neonates of varying GA [13–15]. The PIPP-R includes 2 physiological (heart rate, oxygen saturation), 3 behavioral (brow bulge, eye squeeze, nasolabial furrow) and 2 contextual (GA, behavioral state) variables known to modify pain responses**.** Throughout the treatment intervention, physiological and behavioral/facial indicators of pain intensity were collected using an infant monitoring system developed and used extensively by the research team over the past decade. The research nurse placed pulse oximetry probes on the neonate to record heart rate and oxygen saturation continuously, and positioned a digital video recorder to capture facial movements. Electronic event markers synchronized all physiological and behavioral data, and demarcated the 4 phases of the treatment intervention.

Two trained coders, blinded to group allocation and study purpose, viewed the physiological and behavioral data captured by the infant monitoring system, and coded neonates’ pain intensity using the PIPP-R. An inter-rater reliability > 0.9 was achieved on a random sample of 5 neonates, early in the study and with each 25% of data collected.

The secondary outcome was frequency of a priori specified adverse event/tolerance criteria (heart rate > 240 beats/min or heart rate < 80 beats/min for > 20 s; oxygen saturation < 80% for > 20 s; no spontaneous respirations for > 20 s; and choking/gagging). Adverse event data were collected by the research nurse during the intervention. The research nurse kept a record of ‘rescue doses’ administered (i.e., additional doses of sucrose given on direction of the nurse caring for the neonate, if the neonate became overly distressed during the procedure).

### Statistical analyses

We estimated a sample size of 71 neonates per group (total sample size of 213). The sample size calculation accounts for multiple testing due to 3 intervention groups, and is based on a type I error probability of 5%, a power of 80%, and a smallest minimally clinically significant difference of 1 on the PIPP-R with a standard deviation (SD) of 2. Consistent with previous research, this minimally clinically significant difference was justifiable given the lack of a treatment control in this study versus preceding studies [16]. To account for potential missing data (e.g., equipment failure), we increased the sample size by 15% to 245. Analysis of covariance models adjusting for GA and study site examined between group differences in PIPP-R scores.

## Results

### Randomization and demographic characteristics

**Fig. 1:**
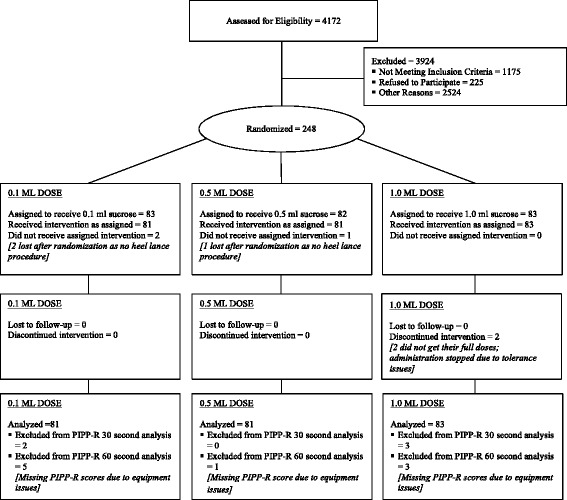
Consort flow diagram of all neonates in participating NICUs screened for eligibility and randomized to sucrose intervention groups. Reasons for exclusion included not meeting inclusion criteria, refusals to participate, and other reasons [e.g., exclusion criteria, medical refusal (palliative care, social issues, and multiple research studies), isolation precautions, and researcher or parents unavailable for consent discussion]

The trial profile is presented in Fig. 1. Of the 4172 neonates screened for eligibility, 248 were enrolled and randomly allocated to Group 1, 2 or 3. Three neonates were excluded following randomization, as they did not undergo a heel lance, leaving 245 for the outcomes analyses. Demographic characteristics in all 3 groups were adequately matched (Table 1). These included GA at birth, days since birth, birth weight, sex, severity of illness assessed using the Score for Neonatal Acute Physiology Perinatal Extension-II (SNAPPE-II) [17, 18], number of prior painful procedures, number of previous doses of sucrose, and concurrent use of non-pharmacologic pain strategies. As standard care in each unit included parent-initiated non-pharmacologic strategies (e.g., swaddling, skin-to-skin/kangaroo care, and breastfeeding) we could not ethically disallow these interventions during the painful procedure. However, there was no difference in the use of parent-initiated pain strategies across groups (Table 1). All neonates were offered a pacifier for non-nutritive sucking following sucrose administration. Overall 204/ 245 (83.2%) sucked on the pacifier, while the remainder refused or did not receive the pacifier due to medical considerations (e.g., intubated, or not tolerated well). We noted a discrepancy between the number of painful procedures documented and the number of sucrose doses documented since birth. Information on non-pharmacologic interventions was often not available in the neonates’ medical records; therefore, it was difficult to discern if the discrepancy was an administration or documentation issue.

**Table 1:** Demographic characteristics of the sucrose intervention groups

	Intervention		
	0.1 ml n = 81	0.5 ml *n* = 81	1.0 ml *n* = 83
Sex, n (%)			
-Female	44 (54.3)	32 (39.5)	41 (49.4)
-Male	37 (45.7)	49 (60.5)	42 (50.6)
Gestational age in weeks, mean (SD)	32.6 (4.2)	32.5 (4.1)	32.7 (4.1)
Weight in grams, mean (SD)	2002.3 (859.5)	1933.0 (927.0)	2055.5 (886.0)
Day of life, median (interquartile range)	6 (4 to 9)	7 (4 to 10)	6 (4 to 9)
Birthplace, n (%) (55 missing)			
-Inborn	31 (52.5)	33 (51.6)	40 (59.7)
-Outborn	28 (47.5)	31 (48.4)	27 (40.2)
SNAPPE-II score on admission, median (interquartile range)	5.0 (0 to 19)	5.0 (0 to 18)	8.0 (0 to 18)
Number of painful procedures since birth, median (interquartile range)	22 (14 to 34)	23 (15 to 37)	23 (13 to 40)
Number of sucrose doses since birth, median (interquartile range)	5 (2 to 8)	5 (3 to 9)	6 (3 to 9)
Use of concurrent non-pharmacologic pain strategies, n (%)	27 (33.3)	31 (35.2)	30 (34.1)

SNAPPE-II scores range from 0 to 158. Higher scores indicate greater severity of illness

### Pain intensity

The mean pain intensity [SD] PIPP-R scores at 30 s post heel lance (Group 1 6.8[3.5]; Group 2 6.8[3.2]; Group 3 6.7[3.4]) were not statistically different after adjusting for GA and research site (F[6233] = 0.01, *P* = .97; Table 2). Similarly, there were no significant differences in mean PIPP-R scores between groups at 60 s (F [2229] = 0.10, *P* = .93; Table 2). Mean pain intensity PIPP-R scores at 30 and 60 s were inversely associated with GA (*P* < .001) and significantly different when stratified by site (*P* < .001; Table 3); therefore both factors were controlled for in the analysis. Mean PIPP-R scores ranged from 6.03 (3.37) for neonates > 36 weeks GA to 9.07 (4.00) for neonates < 28 weeks GA at 30 s and 5.70 (3.31) for neonates > 36 weeks GA to 9.43 (4.04) for neonates < 28 weeks GA. No associations were found between pain intensity scores and other demographic characteristics [i.e., SNAPPE-II/ severity of illness on admission, gender, concurrent use of non-pharmacologic pain strategies (e.g. breastfeeding and skin-to-skin care), and number of painful procedures and sucrose doses since birth; Table 3]. Pain intensity scores across the 3 groups equated to mild pain for the majority of neonates (scores of < 7 on the PIPP-R; Table 4).

**Table 2:** Mean pain intensity scores at 30s and 60s post heel lance

	Intervention			*P*
	0.1 ml	0.5 ml	1.0 ml	
PIPP-R 30s	*n = 79*	*n = 81*	*n = 80*	
	Mean (SD): 6.8 (3.5)	Mean (SD): 6.8 (3.2)	Mean (SD): 6.7 (3.4)	0.97
	Min: 0	Min: 1.0	Min: 0	
	Max: 17.5	Max: 16.3	Max: 18.7	
PIPP-R 60s	*n = 76*	*n = 80*	*n = 80*	
	Mean (SD): 7.0 (3.3)	Mean (SD): 6.9 (3.6)	Mean (SD): 6.7 (3.4)	0.93
	Min: 0	Min: 0	Min: 0	
	Max: 17.0	Max: 18.0	Max: 18.7	

PIPP-R scores range from 0 to 21. Higher scores indicate greater pain intensity

**Table 3:** Association of mean pain intensity scores with site and demographic characteristics

	PIPP-R 30 seconds		PIPP-R 60 seconds	
	Mean (SD)	*P*	Mean (SD)	*P*
Site		< 0.001		< 0.001
1	5.68 (3.31)		5.66 (3.26)	
2	7.55 (3.58)		8.09 (4.16)	
3	6.13 (2.31)		6.05 (2.54)	
4	8.21 (3.87)		8.23 (3.55)	
SNAPPE-II score on admission		0.087		0.058
-Median or below (0 to 5)	6.35 (3.06)		6.38 (3.22)	
-Above Median (6+)	7.03 (3.53)		7.17 (3.70)	
Gender	0.31 (3.32)	0.44	−0.05 (3.49)	0.90
Concurrent use of non-pharmacologic pain strategies during heel lance	0.38 (3.31)	0.33	0.10 (3.49)	0.82
	Spearman’s correlation (r_s_)	*P*	Spearman’s correlation (r_s_)	*P*
Gestational age	−0.26	< 0.001	− 0.30	< 0.001
Number of painful procedures	0.07	0.24	0.03	0.61
Number of sucrose doses since birth	0.004	0.95	−0.02	0.74

PIPP-R scores range from 0 to 21. Higher scores indicate greater pain intensity. SNAPPE-II scores range from 0 to 158. Higher scores indicate greater severity of illness

**Table 4:** Frequency of pain intensity scores by severity at 30s and 60s post heel lance

	Intervention			*P*
	0.1 ml	0.5 ml	1.0 ml	
PIPP-R at 30s, n (%)	*n = 79*	*n = 81*	*n = 80*	0.74
-None (0)	2 (2.5)	0 (0.0)	2 (2.5)	
-Mild (1 to 6.9)	40 (50.6)	46 (56.8)	39 (48.8)	
-Moderate (7 to 11.9)	30 (38.0)	27 (33.3)	33 (41.3)	
-Severe (12+)	7 (8.9)	8 (9.9)	6 (7.5)	
PIPP-R at 60s, n (%)	*n = 76*	*n = 80*	*n = 80*	0.97
-None (0)	1 (1.3)	1 (1.3)	2 (2.5)	
-Mild (1 to 6.9)	38 (50.0)	44 (55.0)	41 (51.3)	
-Moderate (7 to 11.9)	29 (38.2)	26 (32.5)	30 (37.5)	
-Severe (12+)	8 (10.5)	9 (11.3)	7 (8.8)	

PIPP-R scores range from 0 to 21. Higher scores indicate greater pain intensity

### Adverse events and rescue doses

There were 5 reported adverse events among 5/245 (2.0%) neonates as defined by the a priori criteria. These events included 3 neonates who gagged/choked, 1 with heart rate < 80 bpm and 1 with oxygen saturation < 80% following sucrose administration. All events resolved spontaneously without medical intervention. The neonate who experienced oxygen saturation < 80%, was repositioned and recovered quickly. There were no significant differences in the proportion of adverse events by sucrose group (*P* = .62); however, a higher proportion of younger neonates experienced an adverse event (6.7% < 29 weeks versus 1.0% 29–42 weeks; *P* = .044).

In 13/245 (5.3%) neonates, the bedside nurse perceived that the intervention was not effective in minimizing pain during the procedure, and the research nurse (at the discretion of the bedside nurse) administered a “rescue” dose of sucrose (amount determined by the unit standard/policy). There was no significant difference in the number of rescue doses by sucrose group (*P* = .33), site (*P* = .070), or GA (*P* = .47).

## Discussion

Oral administration of a very small dose of sucrose (0.1 ml) appears to be equally effective at reducing pain in neonates during a single painful procedure as larger doses. Sucrose administration in the clinical setting was associated with very few adverse events. This trial was more closely aligned with a pragmatic design on the continuum between pragmatic and exploratory trials [19]. Unlike explanatory trials that test interventions under optimal conditions, pragmatic trials are more generalizable; however, they are also more prone to co-intervention.

Although site was controlled for in the primary outcome analyses, there was a difference in PIPP-R scores across sites (Table 3) that may be partially explained by organizational contextual factors that were not controlled for or assessed in the analyses. For example, although we enrolled neonates in the first 30 of days of life and collected information on exposure to painful procedures and sucrose received since birth, it is possible that sucrose administration and documentation practices differed due to clinical practice guidelines or organizational contextual factors (e.g., workload/staff ratios, unit culture, and the research or clinical experience of the bedside nurses) [20]. We also found higher pain scores were associated with more preterm neonates (*P* < .001; Table 3) and they experienced a slightly greater proportion of adverse events (3 versus 2 in neonates > 29 weeks GA), although total numbers were very small. Despite higher pain scores with lower GA, there was no difference in the number of rescue doses across GA, which might be explained by site differences in sucrose administration practices.

We could think of two possible explanations for why PIPP-R scores were significantly higher in the least mature group of neonates: (a) the PIPP-R measure inherently scores younger GA higher, or b) sucrose is less effective in these babies (e.g., they are less able to mount an endogenous opioid response that is the underlying mechanism of action of sweet taste [21]). Differences seen in mean pain intensity were not thought to be due to additional weighting in the PIPP-R measure by GA [< 28 weeks (+ 3), 28–31 weeks and 6 days (+ 2), 32 weeks to 35 weeks and 6 days (+ 1), and ≥ 36 weeks (0)], as there were no corresponding incremental differences seen by GA group. In terms of the latter explanation (b), this needs to be further researched with an adequate sample size of extremely premature neonates (< 28 weeks GA).

Our findings are consistent with past research (primarily in animals) that demonstrated that the analgesic effects of sucrose were primarily mediated by exposure and not dose [10, 22]. Although there was no difference in pain intensity at 30 and 60 s, pain was not fully eliminated during the heel lance procedure. Mean pain intensity scores equated to mild pain (Table 2), or approximately 3/10 if converted to the more common 10-point scale metric. As pain intensity was measured on a continuum, and treatment failure was not defined, the incidence of treatment failure was not determined. However, severe pain could definitely be considered a treatment failure and this occurred in 7.5 to 11.3% of neonates (Table 4) across sucrose doses. These results are similar to systematic reviews of other behavioral interventions, including breastfeeding [23] and skin-to-skin care [24]. Given that the majority of previous studies have used a single procedure, it is uncertain if the wide variably in neonatal pain response is attributed to the intervention or other factors which remain unknown [25]. Future work in the repeated use of interventions is warranted. In the meantime, we would recommend that if the initial dose of sucrose does not appear to be ameliorating the pain that additional rescue doses be provided during the procedure up to a specified amount. We would also recommend that multiple non-pharmacologic strategies be implemented simultaneously including swaddling, facilitated tucking, skin-to-skin/kangaroo care, breastfeeding, and/ or pacifiers.

Knowledge is lacking on the long-term effects of sucrose with repeated administration. Of the studies that have evaluated repeated doses of sucrose [26–30], none have evaluated long-term outcomes of using sucrose for all painful procedures performed throughout the neonate’s stay in the NICU. Johnston [26, 31] reported that 107 preterm infants < 31 weeks GA who were exposed to > 10 doses of sucrose per day in the first 7 days of life, after which time no pain relief was used, were more likely to exhibit poorer attention and motor development on the Neurobehavioral Assessment of Preterm Infants (NAPI) scale in the early months of life. Conversely, Banga [32] reported that of 93 neonates randomized to either repeated doses of sucrose or water for painful procedures for 7 consecutive days, there were no significant differences in NAPI scores or adverse events. Stevens [27] found no statistically significant differences between sucrose plus pacifier, water plus pacifier, or the standard care group on neurobiological risk status outcomes. Future research needs to address the repeated use of minimally effective doses of sucrose on the neurodevelopment of neonates and effectiveness over time.

Approximately 2% of neonates suffered adverse events. These all resolved spontaneously without medical intervention or with minimal caregiver intervention (e.g. positioning). Most adverse events occurred at one site, where the highest proportion of the sickest neonates is cared for, although this is not represented in the study sample. This adverse event rate is consistent with the 2016 Cochrane sucrose review [2]. Although researchers are becoming more vigilant in observing and reporting adverse events, it remains unclear how adverse events are reported (i.e., chart review is considerably different from careful direct observation of every newborn infant who is receiving the intervention).

A few study limitations need mention. Pain intensity did not differ significantly between the 30 and 60-s time points. Although these time intervals have been used in multiple research studies of acute procedural pain, they are arbitrary and designed based on mean behavioral response time; observing neonates for longer periods of time may demonstrate additional responses of less typical responders or other types of responses (e.g. physiologic, cortical). Although there has been significant validation and updating of the PIPP-R measure, there remains no gold standard for measuring pain in infants that may influence the determination of the effectiveness (or lack thereof) of pain relieving interventions. The future, which includes novel strategies for better understanding of the developing cortical pain circuitry, will pave the way for better prevention and treatment of pain in this vulnerable population.

Finally, we were limited by the documentation in the medical records, which may not have included all pain relieving strategies such as sucrose and non-pharmacologic interventions. Although we believe infants should receive some form of intervention for all painful procedures, it is difficult to speculate on whether the discrepancy between number of documented painful procedures and pain-relieving interventions is an administration or documentation issue. As the number of painful procedures included since birth was extensive (e.g., tape removals, bloodwork, injections, vascular access attempts/insertions, NG/OG tube insertions and suctioning, chest tube attempts/insertions, lumbar punctures, eye exams, and urinary catheterizations), it is possible oral sucrose is not routinely administered for each of these types of procedures, depending on unit standards/practices.

## Conclusions

No difference in pain intensity was shown among 3 doses of sucrose during an acute tissue-damaging procedure in hospitalized neonates. The 0.1 ml of 24% sucrose dose was the minimally effective dose that can be recommended for use out of the 3 doses most commonly reported to be effective in previous research. Subsequent study is required to determine the sustained effectiveness of this dose in reducing pain intensity during painful procedures neonates experience in the NICU over time and across GA, and the long-term effects of cumulative sucrose use.

## Abbreviations

GA: Gestational age
NAPI: Neurobehavioral assessment of preterm infants
NICU: Neonatal intensive care unit
PIPP-R: Premature infant pain profile-revised
SD: Standard deviation
SNAPPE-II: Score for neonatal acute physiology perinatal extension- II

## References

[1] Bueno M, Yamada J, Harrison D, Khan S, Ohlsson A, Adams-Webber T (2013). A systematic review and meta-analyses of nonsucrose sweet solutions for pain relief in neonates. Pain Res Manage.

[2] Stevens B, Yamada J, Ohlsson A, Haliburton S, Shorkey A (2016). Sucrose for analgesia in newborn infants undergoing painful procedures. Cochrane Database Syst Rev.

[3] Lee GY, Yamada J, Kyololo O, Shorkey A, Stevens B (2014). Pediatric clinical practice guidelines for acute procedural pain: a systematic review. Pediatrics.

[4] Taddio A, Yiu A, Smith RW, Katz J, McNair C, Shah V (2009). Variability in clinical practice guidelines for sweetening agents in newborn infants undergoing painful procedures. Clin J Pain.

[5] Bellieni CV, Johnston CC (2016). Analgesia, nil or placebo to babies, in trials that test new analgesic treatments for procedural pain. Acta Paediatr.

[6] Campbell-Yeo M (2016). ‘First, do no harm’--the use of analgesia or placebo as control for babies in painful clinical trials. Acta Paediatr.

[7] Harrison D, Bueno M, Yamada J, Adams-Webber T, Stevens B (2010). Analgesic effects of sweet-tasting solutions for infants: current state of equipoise. Pediatrics.

[8] Randomize.net. A comprehensive internet-based randomization service for clinical trials. http://www.randomize.net (2015). Accessed 11 Apr 2017.

[9] Stevens B, Johnston C, Franck L, Petryshen P, Jack A, Foster G (1999). The efficacy of developmentally sensitive interventions and sucrose for relieving procedural pain in very low birth weight neonates. Nurs Res.

[10] Blass EM, Shide DJ (1994). Some comparisons among the calming and pain-relieving effects of sucrose, glucose, fructose and lactose in infant rats. Chem Senses.

[11] Pillai Riddell RR, Racine NM, Gennis HG, Turcotte K, Uman LS, Horton RE, et al. Non-pharmacological management of infant and young child procedural pain. Cochrane Database Syst Rev. 2015;(12):Cd006275. 10.1002/14651858.CD006275.pub3 PMC6483553 26630545

[12] Ohlsson A, Shah PS. Paracetamol (acetaminophen) for prevention or treatment of pain in newborns. Cochrane Database Syst Rev. 2015;(6):Cd011219. 10.1002/14651858.CD011219.pub2 26110914

[13] Gibbins S, Stevens BJ, Yamada J, Dionne K, Campbell-Yeo M, Lee G (2014). Validation of the premature infant pain profile-revised (PIPP-R). Early Hum Dev.

[14] Stevens BJRNP, Gibbins SRNP, Yamada JRNP, Dionne KRNMN, Lee GRNM, Johnston CRNDF (2014). The premature infant pain profile-revised (PIPP-R): initial validation and feasibility. Clin J Pain.

[15] Lee GY, Stevens BJ, McGrath PJ, Stevens BJ, Walker SM, Zempsky WT (2014). Neonatal and infant pain assessment. Oxford textbook of paediatric pain.

[16] Campbell-Yeo ML, Johnston CC, Joseph KS, Feeley N, Chambers CT, Barrington KJ (2012). Cobedding and recovery time after heel lance in preterm twins: results of a randomized trial. Pediatrics.

[17] Harsha SS, Archana BR (2015). SNAPPE-II (score for neonatal acute physiology with Perinatal extension-II) in predicting mortality and morbidity in NICU. J Clin Diagn Res.

[18] Richardson DK, Corcoran JD, Escobar GJ, Lee SK (2001). SNAP-II and SNAPPE-II: simplified newborn illness severity and mortality risk scores. J Pediat.

[19] Patsopoulos NA (2011). A pragmatic view on pragmatic trials. Dialogues Clin Neurosci.

[20] Estabrooks CA, Squires JE, Hutchinson AM, Scott S, Cummings GG, Kang SH (2011). Assessment of variation in the Alberta context tool: the contribution of unit level contextual factors and specialty in Canadian pediatric acute care settings. BMC Health Ser Res.

[21] Johnston CC, Fillion F, Campbell-Yeo M, Goulet C, Bell L, McNaughton K, Byron J, Aita M, Finley GA, Walker CD (2008). Kangaroo mothercare diminishes pain from heel lance in very preterm neonates: a crossover trial. BMC Pediatr.

[22] Anseloni VC, Weng HR, Terayama R, Letizia D, Davis BJ, Ren K (2002). Age-dependency of analgesia elicited by intraoral sucrose in acute and persistent pain models. Pain.

[23] Shah PS, Herbozo C, Aliwalas LL, Shah VS (2012). Breastfeeding or breast milk for procedural pain in neonates. Cochrane Database Syst Rev.

[24] Johnston C, Campbell-Yeo M, Fernandes A, Inglis D, Streiner D, Zee R. Skin-to-skin care for procedural pain in neonates. Cochrane Database Syst Rev. 2014;(1):Cd008435. 10.1002/14651858.CD008435.pub2 24459000

[25] Cignacco E, Denhaerynck K, Nelle M, Buhrer C, Engberg S (2009). Variability in pain response to a non-pharmacological intervention across repeated routine pain exposure in preterm infants: a feasibility study. Acta Paediatr.

[26] Johnsston CC, Filion F, Snider L, Majnemer A, Limperopoulos C, Walker CD, et al. Routine sucrose analgesia during the first week of life in neonates younger than 31 weeks’ postconceptional age. Pediatrics. 2002;110(3):523-8. 10.1542/peds.110.3.523 12205254

[27] Stevens B, Yamada J, Beyene J, Gibbins S, Petryshen P, Stinson J (2005). Consistent management of repeated procedural pain with sucrose in preterm neonates: is it effective and safe for repeated use over time?. Clin J Pain.

[28] Gaspardo CM, Miyase CI, Chimello JT, Martinez FE, Martins Linhares MB (2008). Is pain relief equally efficacious and free of side effects with repeated doses of oral sucrose in preterm neonates?. Pain.

[29] Taddio A, Shah V, Atenafu E, Katz J (2009). Influence of repeated painful procedures and sucrose analgesia on the development of hyperalgesia in newborn infants. Pain.

[30] Harrison D, Loughnan P, Manias E, Gordon I, Johnston L (2009). Repeated doses of sucrose in infants continue to reduce procedural pain during prolonged hospitalizations. Nurs Res.

[31] Johnston CC, Filion F, Snider L, Limperopoulos C, Majnemer A, Pelausa E (2007). How much sucrose is too much sucrose?. Pediatrics.

[32] Banga S, Datta V, Rehan HS, Bhakhri BK (2016). Effect of sucrose analgesia, for repeated painful procedures, on short-term neurobehavioral outcome of preterm neonates: a randomized controlled trial. J Trop Pediat.

